# Dissociation in relation to other mental health conditions: An exploration using network analysis

**DOI:** 10.1016/j.jpsychires.2020.08.023

**Published:** 2021-04

**Authors:** Emma Černis, Robin Evans, Anke Ehlers, Daniel Freeman

**Affiliations:** a University of Oxford Department of Psychiatry, Warneford Hospital, Oxford, OX3 7JX, UK; b University of Oxford Department of Statistics, 24-29 St Giles', Oxford, OX1 3LB, UK; c Oxford Centre for Anxiety Disorders and Trauma, Department of Experimental Psychology, University of Oxford, The Old Rectory, Paradise Square, Oxford, OX1 1TW, UK; d Oxford Health NHS Foundation Trust, Warneford Hospital, Oxford, OX3 7JX, UK

**Keywords:** Dissociation, Psychosis, Network analysis, Psychopathology, Bayesian inference, Directed acyclic graphs

## Abstract

Dissociative experiences, traditionally studied in relation to trauma and PTSD, may be important phenomena across many different psychological conditions, including as a contributory causal factor for psychotic experiences. In this study, the aim was to explore, using network approaches, how dissociative experiences taking the form of a Felt Sense of Anomaly (FSA) relate to both common mental health conditions and psychotic experiences. 6941 individuals from the general population completed online assessments of FSA-dissociation, post-traumatic stress symptoms (PTSS), anxiety, depression, insomnia, worry, distress tolerance, hallucinations, grandiosity, paranoia, and cognitive disorganization. An undirected partial correlation network analysis was used to explore the network structure, then Bayesian inference with Directed Acyclic Graphs (DAGs) was used to identify potential directions of relationships between dissociation and mental health symptoms. Dissociation was found to be highly connected in both network models. Both networks found direct relationships between dissociation and hallucinations, grandiosity, paranoia, cognitive disorganization, anxiety, depression, and PTSS. In the DAGs analysis, the direction of influence between dissociation and hallucinations, PTSS, anxiety and depression was unclear, however it was found to be probable that dissociation influences paranoia (97.66% of sampled DAGs found the direction dissociation to paranoia, versus 2.34% finding the reverse direction), cognitive disorganization (99.74% vs. 0.26%), and grandiosity (93.49% vs. 6.51%). Further, dissociation was found to be a probable influence of insomnia and distress tolerance via indirect pathways. In summary, dissociation is connected to many mental health disorders, and may influence a number of presentations, particularly psychotic experiences. The importance of dissociation in mental health may therefore currently be under-recognised.

## Highlights

• Dissociation is highly connected to other mental health presentations.
• Dissociation has a strong relationship with hallucinations.
• The direction of effect between dissociation and anxiety, depression and trauma symptoms is unclear.
• Dissociation likely influences paranoia, grandiosity and cognitive disorganization.

## 1 Introduction

Dissociation has been most studied in relation to trauma and PTSD (Ozer et al., 2003). However, dissociation might be best considered a set of transdiagnostic experiences (Schimmenti and Caretti, 2014) that are common (Loewenstein, 2018), and clinically significant in their own right (Lyssenko et al., 2017). Dissociation has been linked with depression (Baker et al., 2007), anxiety (Evren et al., 2008), panic disorder (Fikretoglu et al., 2007), paranoia (Černis et al., 2014), worry (Freeman et al., 2013), and sleep difficulties (Giesbrecht and Merckelbach, 2004). However, despite hypotheses that ‘*dissociation may accompany almost every psychiatric disorder and may influence their phenomenology as well as response to treatment*’ (p.172; Şar, 2014), dissociation remains under-recognised (Loewenstein, 2018). This may be exacerbated by a lack of clarity about the role of dissociation in mental health. In this study, we set out to explore how dissociation may fit within a wide range of mental health symptoms.

### 1.1 Dissociation

The exact definition of the term ‘dissociation’ has been subject to ongoing debate: it has been used to refer to a broad range of experiences, which some argue lacks a unifying ‘single, coherent referent’ (Cardeña, 1994). The current study uses the definition of Černis et al. (in prep.) who identified from patient interviews and systematic review a subset of dissociative experiences sharing the phenomenological common denominator of a ‘felt sense of anomaly’ (FSA). This subjective feeling of ‘strangeness’ (Černis et al., 2020) can take various forms (such as unfamiliarity or detachment) and can occur in relation to various domains, such as one's body, emotions, or external environment.

### 1.2 Dissociation and mental health presentations

Arguments have been made both for dissociation having a causal role in other mental health symptoms and for dissociation resulting from other symptoms. For example, sleep difficulties are one of the key ‘transtheoretical’ factors highlighted by Lynn et al. (2019) as a potential cause of dissociation. This view builds on work demonstrating that experimentally restricted sleep led to increases in dissociative experiences (van Heugten-van der Kloet et al., 2015). Further, affective symptoms, such as anxiety and depression have been found to be predictors for dissociation (Evren et al., 2008). By contrast, positive psychotic symptoms – such as delusions – have been suggested to result from dissociation: Garety et al. (2001) and Freeman (2016) implicate ‘anomalous experiences’ in the formation of these difficulties. However, this causal relationship, and many others across mental health are presently unexplored.

### 1.3 Network analyses

Arising from the network approach to conceptualising mental health presentations (Borsboom, 2017), network analyses have grown in popularity over the past decade as a method for exploring and visualising complex systems of symptoms (Robinaugh et al., 2019). Estimated network psychometrics (Epskamp et al., 2016) model the covariance and pairwise interactions between variables in a network, and can be plotted graphically to visualise these statistical relationships as ‘edges’ between variables (‘nodes’) in order to ‘uncover data patterns’ at the ‘initial stages of phenomena detection’ (Robinaugh et al., 2019). The exploratory value of network analysis methods has seen them rise in popularity in empirical studies. Whilst the majority of these have used ‘undirected’ partial correlation networks, several recent studies have used Bayesian inference with Directed Acyclic Graphs (DAGs) (Bird et al., 2018; Kuipers et al., 2018; Moffa et al., 2017). Unlike undirected networks, DAGs estimate and incorporate information about the likely direction of the conditional dependence relationships between variables. Researchers have begun using this information to make hypotheses about causal relationships. Although there are several caveats to using DAGs in this way – particularly in mental health (Dawid, 2010), interpreted cautiously, this exploratory method can also be highly valuable where it may be helpful to ‘visualise and quantify complex dependencies in the data’ (Robinaugh et al., 2019).

### 1.4 The current study

Network analyses, therefore, may be helpful to explore dissociation. To date, only Schimmenti and Şar (2019) have applied these methods specifically to dissociation: to visualise relationships between items of the Dissociative Experiences Scale. Like that study, the current study uses two network analyses: an undirected network to estimate the conditional associations between variables, and DAGs to hypothesise the possible directions of these relationships. Undirected analyses are easily parameterised and well-identified, giving a sufficiently robust estimation of the true underlying structure of relationships between variables, whilst DAGs are more difficult to identify but are valuable for hypothesis-generation.

The relationships explored here will be those between dissociation and other mental health presentations: post-traumatic stress, depression, anxiety, worry, insomnia, paranoia, grandiosity, hallucinations and cognitive disorganization. Distress tolerance was also included in the analysis as a psychological process, since the leading theoretical view is of dissociation as a way of coping with distress (Dorahy, 2006).

## 2 Methods

### 2.1 Design and participants

The design was an online cross-sectional self-report questionnaire study, with ethical approval granted by the University of Oxford Central University Research Ethics Committee (ref: R57488/RE002). The study was carried out in accordance with the Declaration of Helsinki.

Participants were recruited via social media, the majority via Facebook adverts. A general population sample was considered appropriate since all variables are considered to exist along a continuum of severity and therefore expected to be present, albeit at lower levels, in this group. Advertisements were titled “*Mapping dissociation in mental health*” and stated that questionnaires concerned “*common thoughts and feelings*”. The information sheet described dissociation as “*strange feelings and experiences such as ‘spacing out’, feeling ‘unreal’, or feeling detached from the world around you*”. Inclusion criteria were deliberately broad: any adult (age 18 years or over). There were no exclusion criteria.

During the recruitment period (May 24th, 2018 to July 23rd, 2018), 13,186 responses were recorded by Qualtrics (2018). 144 (1.09%) did not consent to participate, and 307 (2.33%) indicated consent but left the survey without continuing to the first page of measures. After removing participants who did not meet the inclusion criterion, or had high levels of missing data (greater than 20% in any of the measures), a sample of 6941 was obtained for the DAGs analysis.

Of the 6941 participants, the majority were female (87.2%, n = 6050; male: n = 646; other: n = 183) and White (93.7%; n = 6412). The mean age of the sample was 40.3 years (SD = 15.7), with a range from 18 to 86. Additionally, 85.5% of the sample answered “yes” to the question “*have you ever experienced mental health difficulties?*“.

### 2.2 Procedures

Informed consent and assessment were both carried out online using Qualtrics (2018). The questionnaire landing page contained the participant information sheet and statements regarding informed consent, as per British Psychological Society (2017) guidelines for ethical online research. The survey was accessible on desktop and mobile web browsers.

After acknowledging the consent statements, participants were shown seven self-report measures. Unfinished surveys were retrieved automatically after a week of inactivity and added to the dataset.

### 2.3 Measures

Cronbach's alphas for each scale are shown in Table 1. All scales demonstrated ‘good’ or ‘excellent’ internal consistency in this sample.

**Table 1:** Showing the means and standard deviations of the sample for each scale in the survey.

Scale	Sample mean (SD)	Cronbach's alpha for this sample	Scale min – max score	Scale caseness cut-off score
Černis Felt Sense of Anomaly scale	49.74 (32.86)	0.98	0–160	–
Hospital Anxiety and Depression Scale: *Anxiety*	11.17 (4.83)	0.87	0–21	8
Hospital Anxiety and Depression Scale: *Depression*	7.63 (4.69)	0.85	0–21	8
Penn State Worry Questionnaire	57.43 (15.71)	0.95	16–80	45
Insomnia Severity Index	11.90 (6.33)	0.88	0–28	10
Specific Psychotic Experiences Questionnaire: *Paranoia*	23.74 (18.08)	0.95	0–75	–
Specific Psychotic Experiences Questionnaire: *Hallucinations*	7.21 (8.64)	0.91	0–45	–
Specific Psychotic Experiences Questionnaire: *Grandiosity*	3.11 (3.66)	0.86	0–40	–
Specific Psychotic Experiences Questionnaire: *Cognitive disorganization*	7.09 (3.20)	0.84	0–11	–
Post-Traumatic Symptom Disorder Checklist	29.62 (20.09)	0.95	0–80	33
Distress Tolerance Scale	45.74 (14.58)	0.92	15–75	–

NB: Cronbach's alphas above 0.8 are considered to indicate ‘good’ internal consistency, and above 0.9 ‘excellent’.

#### 2.3.1 Černis felt sense of anomaly scale (ČEFSA; Černis et al., in prep.)

This scale measures dissociation in terms of “a felt sense of anomaly” (FSA). The scale comprises 40 items that assess eight factors. The factors are: anomalous experiences of the self (“I feel like a stranger to myself”), of the body (“My body feels numb”), and of emotion (“I don't fully experience emotions”), and altered senses of familiarity (“Places that I know seem unfamiliar”), connection (“I feel disconnected from the world around me”), agency (“I freeze, unable to do anything”), and of reality (“The world seems like it is fake”). The eighth factor is a ‘global FSA’ scale (“Things seem strange”). All items are rated in reference to the past two weeks on a Likert scale from “0, Never” to “4, Always”. Higher scores indicate higher levels of dissociative experiences involving FSA. In this sample, the internal consistency of the scale was excellent (Cronbach's alpha = 0.98).

#### 2.3.2 The hospital anxiety and depression scale (HADS; Zigmond and Snaith, 1983)

The HADS is a 14-item scale comprising two subscales assessing anxiety and depression in the past week. Items such as “I feel tense or ‘wound up’” (anxiety) and “I feel as if I am slowed down” (depression) are rated on a 0 to 3 scale. Higher scores indicate more severe anxiety or depression.

#### 2.3.3 Penn state worry questionnaire (PSWQ; Meyer et al., 1990)

The PSWQ comprises 16 items assessing worry (“I have been a worrier all my life”), rated on a 5-point Likert scale from “1 Not at all typical of me” to “5 Very typical of me”. Higher scores indicate higher levels of worry. Respondents are instructed to consider the past two weeks.

#### 2.3.4 Insomnia severity Index (ISI; Morin, 1993)

The ISI assesses insomnia and its impact on day-to-day life over the past two weeks using seven items scored on a 0 to 4 Likert scale. Higher scores indicate more problematic sleep.

#### 2.3.5 Specific psychotic experiences questionnaire (SPEQ; Ronald et al., 2013)

The SPEQ comprises four scales which each assess a key psychotic experience: paranoia, hallucinations, grandiosity, and cognitive disorganization.

The *paranoia* and *hallucinations* scales ask respondents to rate how frequently they have recently had particular thoughts (e.g. ‘How often have you thought: I am under threat from others’) or experiences (e.g. ‘How often do you: hear noises or sounds when there is nothing about to explain them?‘), respectively, using a six-point Likert scale (‘not at all’ to ‘daily’). The *grandiosity* scale asks respondents how much they agree with the statements (e.g. ‘I have a special mission’) for the past month, on a four-point Likert scale (‘not at all’ to ‘completely’). Finally, the *cognitive disorganization* scale also assesses the past month, asking for ‘yes/no’ responses to items such as ‘are you easily confused if too much happens at the same time?‘. For all scales, higher scores indicate more severe psychotic experiences.

#### 2.3.6 Post-traumatic symptom disorder checklist (PCL-5; Weathers et al., 2013)

To assess trauma symptoms over the past month, the PCL-5 contains 20 items such as “feeling very upset when something reminded you of the stressful experience”, rated on a five-point Likert scale from “0 not at all” to “4 extremely”. Participants were asked to rate “the most upsetting event” they had experienced, which included traumatic and other upsetting life events as indicated via selecting from a list including “end of a relationship”, “natural death of a significant other”, and “severe accident”. Higher scores indicate greater post-traumatic symptomatology.

#### 2.3.7 Distress tolerance scale (DTS; Simons and Gaher, 2005)

Using a five-point Likert scale from “1, strongly agree” to “5, strongly disagree”, the DTS measures beliefs and responses to strong emotions, such as “feeling distressed or upset is unbearable to me”. Higher scores indicate less tolerance of strong emotions. Respondents are asked to ‘think of times that you feel distressed or upset’, rather than a specific timeframe when answering.

### 2.4 Statistical analysis

Analyses were conducted in R, version 3.6.3 (R Core Team, 2020). Multiple imputation (using ‘mice’ package) was used for missing data to allow calculation of scale total scores, meaning that all variables were continuous. The proportion of imputed data was very low: less than 0.1% of the data for each scale. Visual inspection of variable histograms revealed a non-normal distribution. Therefore, data were transformed to a normal distribution (‘gaussianized’) using the ‘DAGtools’ (v0.1.001 l) package prior to analysis.

#### 2.4.1 Undirected network

As a first step in exploring the relationships within the data, a Gaussian Graphical model was estimated. These undirected networks can also be visualised using the qgraph package such that the strength of the conditional relationship between variable (node) pairs (once all other variables have been conditioned upon) are represented by the weight of the edge between them (i.e. line width and shading). In this way, the absence of an edge between two nodes indicates conditional independence between those two variables, once all other variables have been conditioned upon.

Undirected networks are more easily parameterised and make no assumptions regarding the direction of relationships between nodes. These qualities are helpful in estimating the true underlying structure of the data (‘identification’), as it eliminates the problem of Markov equivalence: one of the key limitations of DAGs. Markov equivalence refers to cases where the same set of conditional independence parameters could be represented by numerous different configurations between nodes, including contradictory information about directions of effect. Without additional knowledge, it would be unclear which model most closely represented the real-world situation from which the data was collected. Undirected networks also do not assume acyclicity (that nodes do not have reciprocal relationships – an assumption that is particularly unlikely to be true in mental health research). These networks can therefore provide a more robust estimate of the true structure of the relationships within the data than DAGs.

To estimate and visualise the undirected network, the packages ‘bootnet’ (v1.3) and ‘qgraph’ (Epskamp et al., 2012) were used. Due to the large sample size, ggmModSelect was used to fit a Gaussian graphical model to the data via stepwise model selection in order to optimise model fit. The argument ‘principal direction’ was used during estimation to recode all variables in the same direction: i.e. in order that higher scores for all variables indicated more severe negative outcomes. Non-parametric bootstrapping (5000 bootstraps) was used to assess the accuracy and stability of the estimated network (*Supplementary Material*). In the final graph, positive partial correlations are shown by a blue and negative correlations by a red line. The strength of the pairwise partial correlations between nodes is indicated in both cases by the weight of the edge.

#### 2.4.2 Bayesian inference with directed acyclic graphs

An advantage of the Bayesian inference with DAGs method is that it can hypothesise the direction of direct and indirect pathways between nodes. This is valuable in a field where much is unknown. In a DAG, if variable B is directly dependent on variable A, this is indicated by a directed edge (→) between the nodes, such that ‘A→B’. This can be interpreted both as “controlling for other parents of B, B is dependent upon A”, and “A is a direct cause of B”. As in undirected networks, nodes not joined by an edge can be interpreted as being (conditionally) independent from each other given some subset of the other variables.

There are important limitations to this method, in addition to the problems of assumed acyclicity and Markov equivalence discussed above. Firstly, it is important to note that mathematical independence is not necessarily indicative of real-world causal independence (Dawid, 2010) and therefore these networks require careful interpretation: they should be considered hypothesis-generating when the true underlying causal structure is unknown. This method also assumes causal sufficiency: that there are no hidden common causes shared by nodes, and that all important causal variables have been included in the network.

An advantage of incorporating Bayesian inference into the DAGs methodology is that it enables the probability of each edge to be estimated. Whilst this does not remove the problems and limitations described above, it does provide further detail with which to better interpret the network. In this analysis, the final graph was calculated by averaging the results of 50,000 sample DAGs, obtained by using the BiDAG package to run the partition Markov Chain Monte Carlo algorithm (Kuipers & Moffa, 2017, Kuipers et al., 2018) for 10 million iterations. The exact method of this analysis and the preceding transformation of the data follows the same protocol as detailed in Bird et al. (2018). For this study, a directed edge (i.e. A→B) in the final graph indicates that this specific direction occurred in over 90% of the sampled DAGs for which that edge was present. Only these cases were considered as sufficient for drawing hypotheses about probable direction of influence. Edges are shown as undirected (i.e. A-B) where the edge was present in over 50% of cases, but the above criterion regarding direction was not met. Causal effects are shown as z-scores with 90% credible intervals (CIs). In practice, a credible interval may be interpreted in a similar manner to a confidence interval, but it is calculated according to the probability distribution given the data. The results of the DAGs analysis therefore provide information both about how often a directed relationship between two variables is found, and the strength of this causal effect.

## 3 Results

Table 1 shows the mean scores for each scale. Generally, scores were within the non-clinical range, with mildly elevated anxiety, worry, and sleep difficulties according to scale cut-offs.

### 3.1 Undirected network

**Fig. 1:**
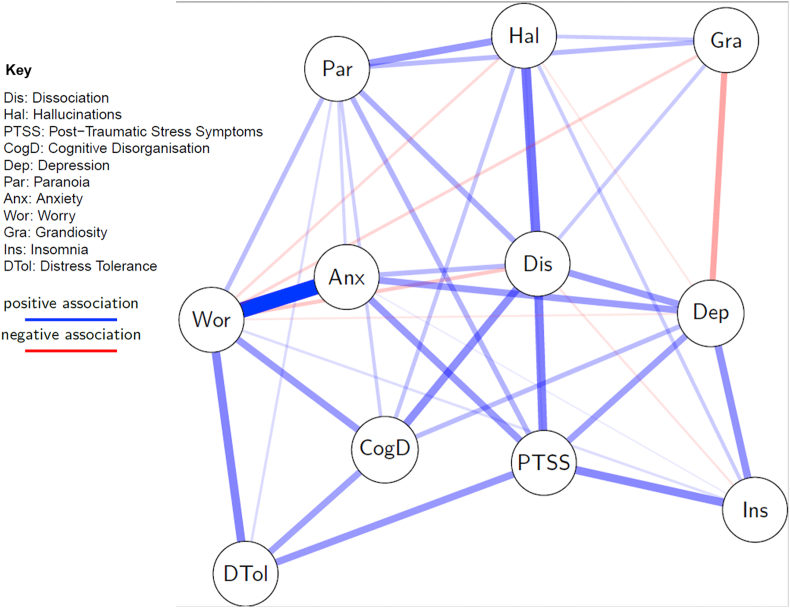
Undirected network graph showing relationships between dissociation, psychotic-like experiences, and other presentations measured in the online survey. Red lines show negative relationships. Blue lines show positive relationships. Greater thickness and colour strength of edges indicates greater edge weight.

Fig. 1 shows the undirected network (see *Supplementary Material* for full details). In summary, dissociation had direct relationships with all variables except distress tolerance. Dissociation was highly central in the network in terms of degree centrality (1.50), closeness (0.0149), and betweenness (14). The stability of centrality estimates was very high (coefficient 0.75 for each) and the accuracy of network estimation (reflected in edge-weight 95% bootstrapped confidence intervals; CIs) was good. The strongest edge in the network was between worry and anxiety (edge-weight = 0.452, CI = 0.431–0.472) – this was statistically significantly stronger than any other edge. The next strongest edges in the network were between dissociation and hallucinations (edge-weight = 0.273, CI = 0.247–0.298), and dissociation and post-traumatic stress symptoms (PTSS; 0.249, CI = 0.195–0.246). The edge-weight between dissociation and hallucinations was not significantly higher than that between dissociation and PTSS but it was significantly higher than all other edges involving dissociation. Dissociation's weakest edges were with grandiosity (0.103, CI = 0.0797–0.127), and insomnia (−0.0574, CI = −0.0913 to −0.0236). An edge was not found between dissociation and distress tolerance.

### 3.2 Directed Acyclic Graphs (DAGs)

Table 2 summarises the results of the DAGs analysis for the relationships between dissociation and all other variables.

**Table 2:** Summarising the average causal effects between dissociation and all other variables.

Causal effects:	Pathway present (direct or indirect) % (2 sf)		Causal effect	90% CI	Direct edge present %	Direct causal effect	90% CI
**Variable to dissociation (i.e. variable causing dissociation)**							
Anxiety		48.85	0.53	0.27–0.64	100	0.43	0.21–0.63
Hallucinations		29.75	0.56	0.37–0.66	100	0.48	0.32–0.66
Depression		27.51	0.51	0.26–0.63	100	0.43	0.24–0.63
Post-traumatic Stress		13.81	0.58	0.40–0.72	100	0.50	0.39–0.70
Worry		13.45	0.32	0.02–0.47	35.51	0.12	0.00–0.47
Insomnia		7.39	0.27	0.02–0.45	22.43	0.03	−0.01–0.22
Grandiosity		6.51	0.18	0.13–0.21	99.69	0.16	0.06–0.21
Paranoia		2.34	0.44	0.20–0.65	100	0.32	0.20–0.59
Distress Tolerance		1.52	−0.26	−0.51–−0.07	100	−0.09	−0.23–−0.06
Cognitive Disorganization		0.26	0.53	0.34–0.67	100	0.30	0.21–0.49
**Dissociation to variable (i.e. dissociation causing variable)**							
Anxiety		51.15	0.51	0.20–0.63	100	0.42	0.17–0.62
Hallucinations		70.25	0.53	0.44–0.58	100	0.50	0.41–0.58
Depression		72.49	0.46	0.24–0.61	100	0.35	0.23–0.61
Post-traumatic Stress		86.19	0.51	0.35–0.71	100	0.33	0.29–0.55
Worry		82.76	0.21	0.01–0.46	4.66	<0.01	0.00–0.00
Insomnia		90.13	0.23	0.06–0.44	4.30	<0.01	0.00–0.00
Grandiosity		93.49	0.19	0.06–0.20	100	0.16	0.13–0.20
Paranoia		97.66	0.46	0.29–0.65	100	0.21	0.17–0.25
Distress Tolerance		98.48	−0.30	−0.50–−0.09	19.48	−0.01	−0.07–0.00
Cognitive Disorganization		99.74	0.44	0.31–0.61	100	0.25	0.22–0.28
**Key:**							
*‘Pathway present’*		The proportion of sampled DAGs which found this pathway.					
*‘Causal effect’*		Average total causal effect when that pathway was present.					
*‘Direct edge present’*		The proportion of DAGs that found direct pathways of those where some pathway was found to be present.					
*‘Direct causal effect’*		Average total causal effect of the direct pathways.					
*CI*		Credible interval.					

#### 3.2.1 Affective symptoms

The table can be interpreted as follows: from anxiety to dissociation, there was a pathway (direct or indirect) present in 48.85% of the 50,000 DAGs sampled – that is, anxiety influences dissociation in 48.85% of sampled graphs. The average strength of the causal effect within these 48.85% of graphs was 0.53 (with a 90% credible interval (CI) 0.27–0.64). In 100% of the 48.85% of DAGs sampled which showed a pathway from anxiety to dissociation, a *direct* edge between anxiety and dissociation was present, and this had an average strength of 0.43 (90% CI = 0.21–0.63). The opposite direction, from dissociation to anxiety, was present in 51.15% of sampled graphs, with average strength 0.51 (CI = 0.20–0.63). Again, 100% of the 51.15% of sampled DAGs showing a pathway from dissociation to anxiety contained a direct pathway. The average strength of this direct pathway was 0.42 (CI = 0.17–0.62). Therefore, there is a significant direct relationship between anxiety and dissociation but no clear direction of influence, as roughly equal proportions of the 50,000 DAGs sampled showed each direction.

Depression and PTSS also show significant direct relationships with dissociation. The direction is unclear in depression (72.49% dissociation influences depression; 27.51% vice versa), but more suggestive with PTSS (86.19% dissociation influences PTSS; 13.81% vice versa). However, neither meets the guideline described above for drawing hypotheses about probable direction of influence (i.e. direction present in at least 90% of sampled DAGs).

#### 3.2.2 Psychotic symptoms

The above results contrast with the results for cognitive disorganization, where 0.26% of the sampled DAGs found an edge from cognitive disorganization to dissociation, but 99.74% showed the opposite, with 100% of these showing a direct pathway with average strength 0.25. This can be interpreted as suggesting that the probability that dissociation contributes to cognitive disorganization is close to 1.

It was found to be highly probable that dissociation also influences two other psychotic experiences (rather than vice versa): 97.66% and 93.49% of sampled DAGs found that dissociation influences paranoia and grandiosity respectively. The exception was hallucinations, where the direction of the relationship was less clear: 70.25% of sampled DAGs found that dissociation influences hallucinations, but the remaining 29.75% found the opposite direction. This proportion is insufficient to draw inferences about direction of effect.

#### 3.2.3 Indirect relationships

Relationships between dissociation and insomnia, worry, and distress tolerance were strongly indicated to follow the direction of dissociation influencing each of these variables (insomnia: 90.13%; worry: 82.76%; distress tolerance: 98.48%). However, the majority of influence came from indirect pathways with very small effect sizes (see Table 3).

**Table 3:** Summarising the indirect pathways between dissociation and worry, insomnia, and distress tolerance and their causal effects.

	Dissociation to Worry: 82.76% of sampled DAGs		Dissociation to Insomnia: 90.13% of sampled DAGs		Dissociation to Distress Tolerance: 98.48% of sampled DAGs	
	Pathway present (% of 82.76%)	Causal effect	Pathway present (% of 90.13%)	Causal effect	Pathway present (% of 98.48%)	Causal effect
Direct pathway	4.66	0.0058	4.30	0.0014	19.48	−0.011
**Indirect pathway via:**						
Anxiety	50.12	0.19	47.44	0.075	49.3	−0.083
Hallucinations	60.94	0.0083	62.58	0.052	69.73	−0.027
Depression	62.72	0.026	64.15	0.107	70.76	−0.036
PTSS	12.22	0.014	12.75	0.033	87.20	0.141
Worry	*Direct pathway*		10.37	0.0038	83.36	−0.064
Insomnia	85.25	0.019	*Direct pathway*		88.23	−0.015
Grandiosity	4.30	<0.001	4.79	<0.001	7.26	–
Paranoia	0.63	0.0012	1.75	<0.001	91.88	−0.036
Distress Tolerance	0.53	<0.001	0.53	<0.001	*Direct pathway*	
Cognitive Disorganization	–	–	0.13	–	96.19	−0.090

#### 3.2.4 Graphical representation

**Fig. 2:**
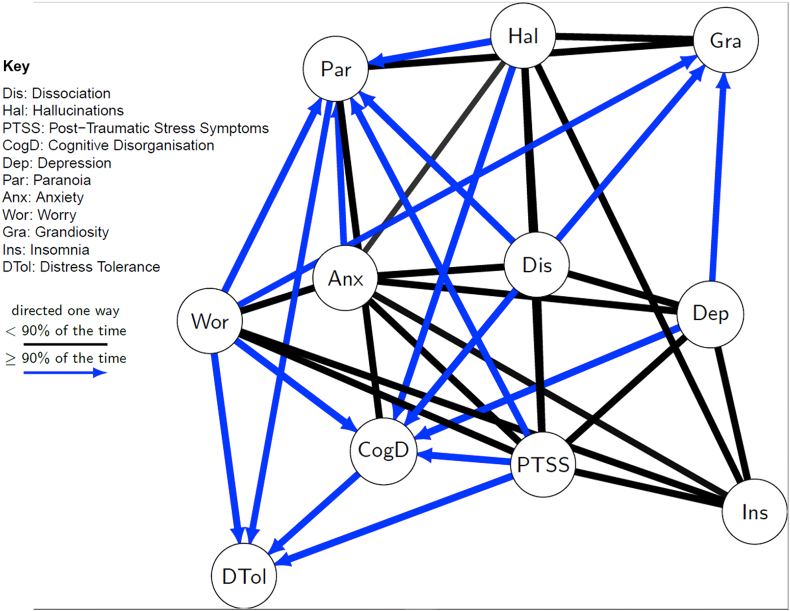
Mixed graph (i.e. with both directed and undirected edges) showing relationships between dissociation, psychotic-like experiences, and other presentations measured in the online survey. Undirected lines (black, no arrows) show relationships that were present in over 50.00% of the 50,000 sampled DAGs. Blue lines with arrowheads show relationships (and the probable direction of causation) where these were present in over 90.00% of the 50,000 sampled DAGs.

Fig. 2 summarises the results of Table 2. Edges are shown when the relationship between the variables was present in over 50.00% of the 50,000 sampled DAGs. Where the edges are directed (blue, with arrowheads), the indicated direction of influence was present in over 90.00% of the DAGs where the edge was present. Therefore, black edges without arrowheads show a relationship where the direction is more ambiguous, and edges with arrowheads indicate a more probable direction (in the direction shown by the arrowhead).

#### 3.3 Comparison of networks

Both networks indicate that dissociation is highly connected to all the other mental health variables, with the exception of distress tolerance. The absence of an edge between dissociation and distress tolerance in the undirected network is supported by the DAGs analysis, where very few direct pathways between the two were found in the 50,000 sampled DAGs.

In terms of the strength of the direct relationships with dissociation, hallucinations and PTSS have the highest edge-weights with dissociation in the undirected network and the strongest direct causal effects in the DAGs analysis. Therefore, both networks find that these variables have robust relationships with dissociation.

For other relationships, the two network models did not yield identical results. For example, whilst the direct causal effects for depression (0.43 influence on dissociation and 0.35 influence from dissociation) and anxiety (0.43 and 0.42) appear similar to those for PTSS (0.50 and 0.33) and hallucinations (0.48 and 0.50) in the DAGs analysis, their edge-weights with dissociation in the undirected analysis do not support this. Anxiety in particular has a significantly lower edge-weight (0.138; CI: 0.112–0.165) than that for depression (0.192; CI:0.164–0.219), which is in turn significantly lower than those for hallucinations (0.273; CI:0.247–0.298) and PTSS (0.249; CI:0.195–0.246) (which were not statistically significantly different). Indirect pathways between these variables may have influenced the results in the undirected network, since anxiety and depression are likely to be highly associated.

Similarly, at first glance, the relationships between dissociation and worry and dissociation and insomnia appear to differ between models. The undirected network model shows a weak negative relationship between dissociation and these two variables (worry: −0.107, CI: 0.135 to −0.0797; insomnia: −0.0574, CI: 0.0913 to −0.0236), whilst the DAGs analysis found small positive effects. However, both models agree that the strength of the direct relationships is low, perhaps too weak to estimate reliably. The DAGs analysis result is influenced by the indirect pathways from dissociation to both variables, which appear robust (i.e. they were found in more than 50% of sampled DAGs, and the indirect effects of dissociation on insomnia exceeds the 90% threshold for drawing tentative conclusions regarding direction of influence).

## 4 Discussion

Dissociation is a relatively neglected area of psychological research and treatment, requiring ‘innovative thinking and research’ (p.171; Şar, 2014). Here, we use state-of-the-art network analysis methods to explore the position of dissociation in the wider constellation of mental health presentations and generate hypotheses for future research. Both networks suggest dissociation is highly connected with other mental health conditions, including post-traumatic stress symptoms (PTSS), anxiety and depression. Further, dissociation is likely to influence paranoia, grandiosity, and cognitive disorganization. This is consistent with existing models of delusions (Maher and Ross, 1984; Garety et al., 2001; Freeman, 2016), and a recent meta-analysis of the relationships between dissociation and psychotic symptoms (Longden et al., 2020).

We wish to highlight some results of particular interest. First, robust relationships between hallucinations and dissociation and between PTSS and dissociation featured in both networks, but in both cases, the direction of influence was unclear. This may indicate that these constructs are interconnected with dissociation either by sharing a common cause not represented in the networks, or through a reciprocal relationship (or both of these). For example, whilst dissociation is often considered a post-traumatic symptom (Loewenstein, 2018), peri-traumatic dissociation has been found to predict later PTSD (Ozer et al., 2003), indicating a more complex relationship. Alternatively, high conceptual overlap may also produce this result. Indeed, of relevance to the result for hallucinations, Moskowitz and Corstens (2008) suggest that auditory hallucinations are a form of dissociation.

Second, the direct relationships from worry to dissociation, and from insomnia to dissociation found in previous experimental manipulation studies (worry: Freeman et al., 2013; insomnia: van Heugten–van der Kloet et al., 2015) were not replicated in this study. Rather, the DAGs analysis was unable to find a clear direction of influence between worry and dissociation, and indicated that there was a high probability of dissociation indirectly influencing insomnia. Further, the undirected network found weak negative relationships between these variables and dissociation. The discrepancy between networks and the contrast with existing literature may be due to several factors: the direct relationships being too weak to be estimated reliably, the influence of other variables within the indirect pathways, and the limitations of these methods for exploring reciprocal relationships. Indeed, for worry, the relationship with dissociation was found to be mediated by anxiety in the DAGs analysis, and Freeman et al. (2013) did find increased anxiety ratings in their worry induction condition. Regarding the result for insomnia, Lynn et al. (2019) suggest that mediation of the relationship between sleep and dissociation by other variables is an important consideration. One such consideration could be negative affect, since Reeve et al. (2018) highlight the mediating role of this in the relationship between insomnia and psychotic symptoms.

Third, the results for dissociation and cognitive disorganization are important conceptually. A robust relationship between the two was found in both networks, with high probability that dissociation influences cognitive disorganization. This suggests that these are distinct concepts and that confusion, loosening of associations, thought disruption, and other hallmarks of cognitive disorganization are not analogous to dissociation, but may result from it.

Finally, analysis strongly supported the interpretation that distress tolerance is indirectly related to dissociation, most likely as a downstream effect. This echoes anecdotal reports that dissociation sometimes helps individuals to better tolerate distress when under extreme stress. However, it does not support the theoretical position that dissociation is selected as a coping strategy as a result of a person's low distress tolerance. Interestingly, a very large proportion of indirect pathways from dissociation to distress tolerance included paranoia and cognitive disorganization. This suggests that the subjective experiences of threat and thought disruption may be particularly likely to overwhelm people's capacity to tolerate distress. Taken together with the results indicating that dissociation influences paranoia, grandiosity and cognitive disorganization, this study implies an important role for dissociation in the symptomatology and lived experience of psychosis.

A limitation of the study is its sampling method: recruitment via Facebook attracted a predominately White female sample who engage with social media and are willing to participate in online research. This may be unrepresentative of the wider population. Further, these findings require replication in a clinical group to test the assumption that subclinical levels of the variables interact in a manner analogous to clinically significant levels.

As previously discussed, there are multiple limitations inherent in the DAGs methodology. Acyclicity is a fundamental assumption of the analysis, therefore DAGs cannot estimate reciprocal relationships and feedback loops which are likely to be important when considering psychological variables. DAGs based on cross-sectional data assume all relevant variables are included and cannot contain information about longitudinal effects between variables. These limitations, and the problems inherent in assuming that mathematical causality reflects real-world causal structure, lead some to argue DAGs are suitable only for describing networks where the underlying causal structure is already known. However, we agree with Robinaugh et al. (2019) that they can ‘*provide valuable but incomplete information about the relationships between symptoms* [nodes]’ (p.6) and that the resulting ‘interesting causal conjectures’ (p.83; Dawid, 2010) may still be valuable – particularly in a field such as dissociation. Furthermore, the similarities between the undirected and DAG networks and the parsimony between our results and the existing literature are heartening and suggest that the ‘interesting causal conjectures’ presented here warrant further exploration with experimental designs. In a field where the underlying causal structure is very much unknown, the value of network analyses is in generating ideas which we hope will invigorate professional interest. This study highlights that dissociation may well have a prominent position in the broad network of psychological disorders, and psychosis in particular, and hence merits greater attention.

## Financial support

The work was supported by the 10.13039/100010269Wellcome Trust via a Clinical Doctoral Fellowship to EČ (grant number 102176/B/13/Z). DF is supported by an 10.13039/501100000272NIHR Research Professorship (NIHR-RP-2014-05-003. AE is funded by the 10.13039/100010269Wellcome Trust (200796) and supported by the 10.13039/501100000272Oxford Health NIHR Biomedical Research Centre and a 10.13039/501100000272NIHR Senior Investigator Award. The views expressed are those of the authors and not necessarily those of the National Health Service, NIHR, or Department of Health.

## CRediT authorship contribution statement

**Emma Černis:** Conceptualization, Data curation, Methodology, Software, Formal analysis, Investigation, Writing - original draft, Project administration, Funding acquisition. **Robin Evans:** Software, Formal analysis, Software, Formal analysis, Validation, Visualization. **Anke Ehlers:** Conceptualization, Writing - review & editing, Supervision, Methodology. **Daniel Freeman:** Conceptualization, Writing - review & editing, Supervision, Methodology.

## Declaration of competing interest

The authors declare no conflicts of interest.
