# Supplementary material for: Dissociation in relation to other mental health conditions: An exploration using network analysis
Source: J Psychiatr Res. 2021 Apr;136:460–7. doi: 10.1016/j.jpsychires.2020.08.023 (PMC8039185; doi:10.1016/j.jpsychires.2020.08.023)
Supplement: Multimedia component 1 [file mmc1.docx]

**Dissociation in relation to other mental health conditions: An exploration using network analysis**

**SUPPLEMENTARY MATERIAL:
Assessing the accuracy and stability of the undirected network**

1. **Assessing the estimated network connections**

To assess the accuracy of the connections in the estimated undirected network, bootstrapped confidence intervals (BCIs) for each edge were calculated using non-parametric bootstrapping (5000 bootstraps) using bootnet (v1.3). Bootstrapped confidence intervals for all edges in the undirected network are shown in Figure s1. This indicates some level of overlap of BCIs between edges in the network (except for that between worry and anxiety which was very strong). Nevertheless, the strongest edges (anxiety-worry; dissociation-hallucinations; dissociation-PTSS) are significantly stronger than the majority of others, and the network appears to be estimated with good accuracy.


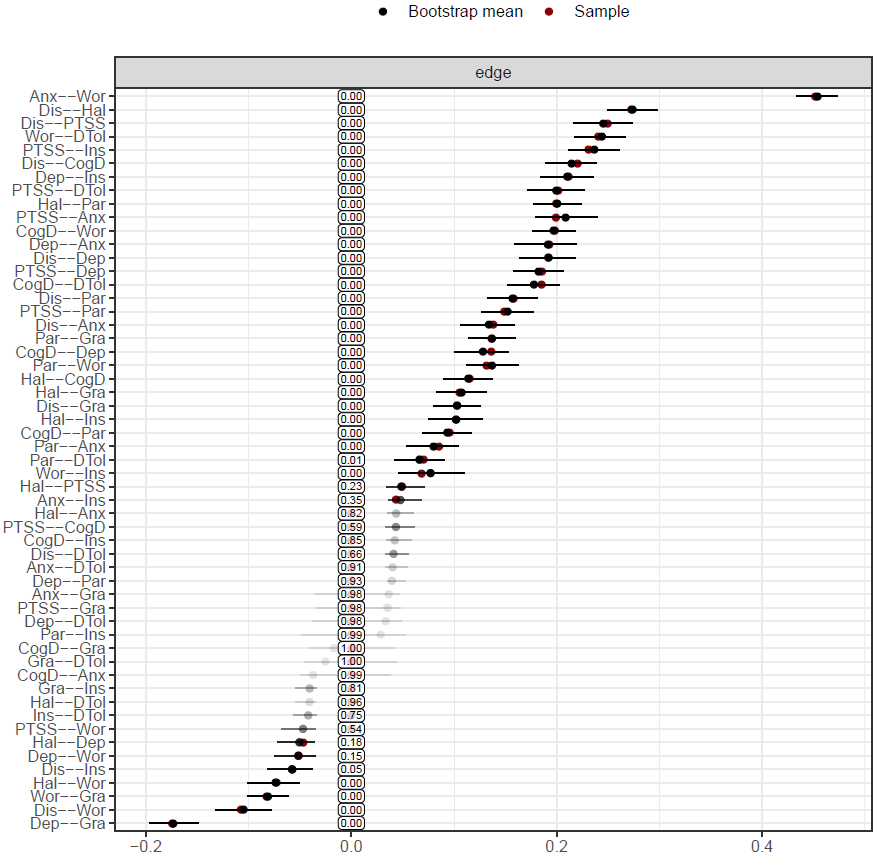
***Figure s1.*** *Showing sample edge-weights and bootstrapped confidence intervals of edge-weights obtained via non-parametric bootstrapping (5000 bootstraps) for all edges in the undirected network*

Table s1 shows the edge-weights and their bootstrapped confidence intervals used to assess the differences in edge-weights for edges between dissociation and all other variables.

| ***Table s1.*** *edge-weights and their bootstrapped confidence intervals for edges between dissociation and each other variable* | | |
| --- | --- | --- |
| **Variable** | **Edge-weight** | **B.C.I.** |
| Hallucinations | 0.273 | 0.247 – 0.298 |
| Post-Traumatic Stress Symptoms | 0.249 | 0.195 – 0.246 |
| Cognitive Disorganisation | 0.220 | 0.195 – 0.246 |
| Depression | 0.192 | 0.164 – 0.219 |
| Paranoia | 0.158 | 0.133 – 0.183 |
| Anxiety | 0.138 | 0.112 – 0.165 |
| Worry | -0.107 | -0.135 – -0.0797 |
| Grandiosity | 0.103 | 0.0797 – 0.127 |
| Insomnia | -0.0574 | -0.0913 – -0.0236 |
| Distress Tolerance | 0 (no edge) | -0.0395 – 0.0395 |

The results in Table s1 indicate that the strongest edge between dissociation and another variable is between dissociation and hallucinations. This edge-weight is not significantly stronger than that between dissociation and post-traumatic stress symptoms (PTSS), but is significantly stronger than all other edges with dissociation.

Edge-weight difference tests for all edges are shown in Figure s.2. This indicates that many edges are statistically significantly different from others.

***Figure s2.*** *Differences between edge-weights: statistically significant differences indicated by a black square, non-significant differences by a grey square*


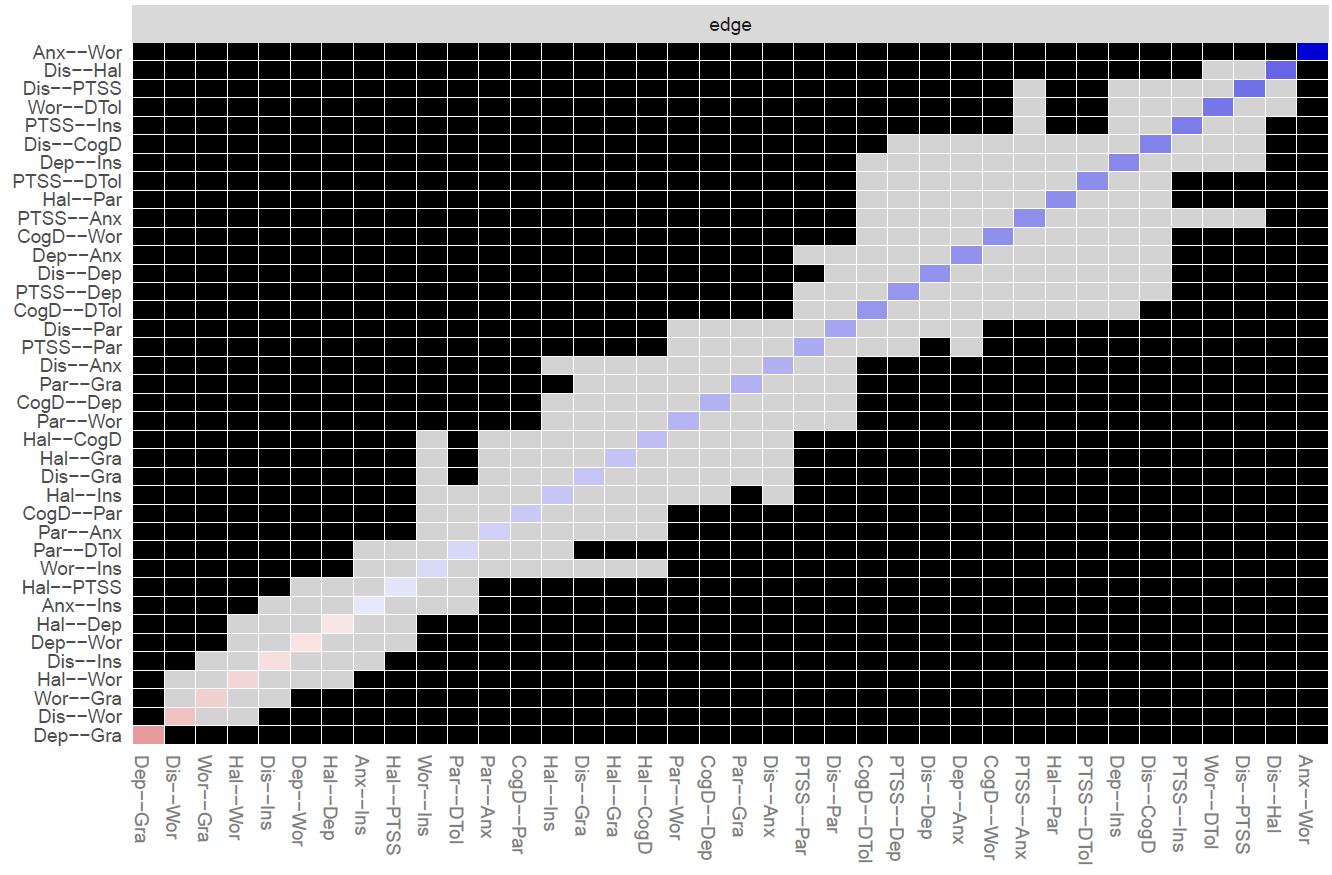


1. **Assessing centrality indices**

Centrality estimates (strength, closeness and betweenness) were calculated for all variables. The results are shown in Table s2 and Figure s3. Tests were carried out to assess for statistically significant differences between centrality estimates:

| ***Table s2.*** *Centrality measures for all variables, with the highest scores shown in bold* | | | |  |
| --- | --- | --- | --- | --- |
| **Variable** | **Strength (Degree)** | **Closeness** | **Betweenness** | |
| Dissociation | **1.50** | **0.0149** | **14** | |
| Worry | **1.40** | 0.0126 | 6 | |
| Post-Traumatic Stress Symptoms | 1.26 | **0.0154** | **20** | |
| Paranoia | 1.03 | 0.0114 | 2 | |
| Insomnia | 0.713 | 0.0110 | 0 | |
| Hallucinations | 0.962 | 0.0112 | 0 | |
| Grandiosity | 0.602 | 0.00943 | 0 | |
| Distress Tolerance | 0.697 | 0.0111 | 0 | |
| Depression | 1.19 | 0.0139 | **12** | |
| Cognitive Disorganisation | 0.950 | 0.0121 | 0 | |
| Anxiety | 1.11 | 0.0135 | 6 | |

*Node strength (degree centrality):* This centrality measure estimates how strongly a node is connected with the network by summing all edges it has with all other nodes. Dissociation has the highest node strength in the network. Difference tests found that it is significantly higher than for PTSS (3^rd^ highest), but not statistically significantly higher than for worry (2^nd^ highest).

*Closeness:* This centrality measure estimates how strongly a node is connected indirectly with the network using information about the shortest path lengths between the node and all other nodes. Dissociation has the second highest closeness score in the network, with the highest being PTSS. Again, there was no statistically significant difference between these two scores. The closeness score for dissociation is statistically significantly higher than that for depression (3^rd^ highest).

*Betweenness:* This centrality measure uses information about the shortest path lengths in the network to determine how often a node is a point along a shortest pathway between other nodes. This indicates how often the node acts as a connector node. Dissociation has the second highest betweenness score of the nodes in the network, with PTSS the highest. Difference tests found no significant difference between the two. The betweenness for dissociation was also not statistically different from that for depression (3^rd^ highest). It was statistically significantly higher than anxiety (4^th^ highest).

The results of difference tests for all centrality estimates for all variables are shown in Figure s4.

***Figure s3.*** *Showing centrality scores for all variables in the network*


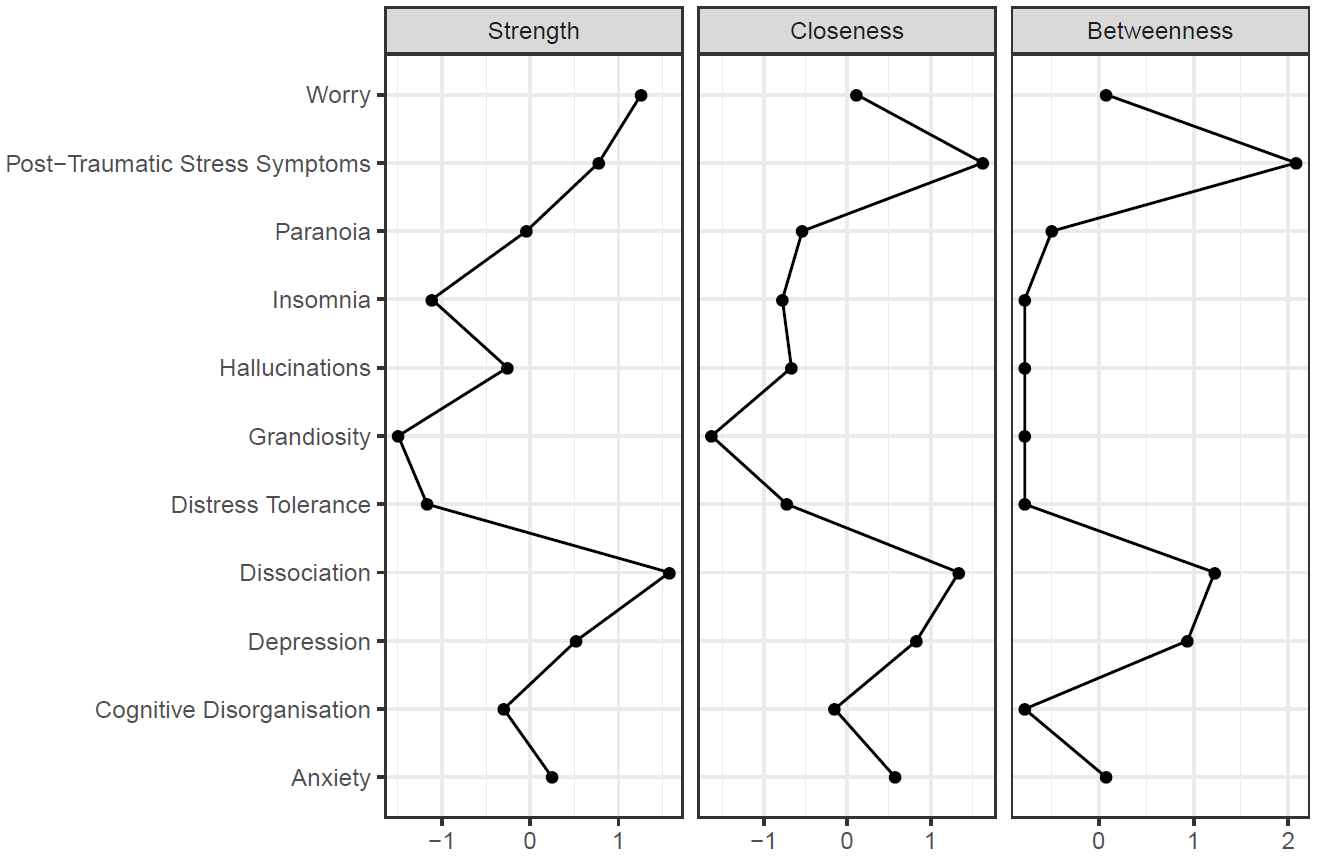


***Figure s4.*** *Differences between centrality estimates: statistically significant differences indicated by a black square, non-significant differences by a grey square.*

*Panel 4a: Strength (degree centrality)*


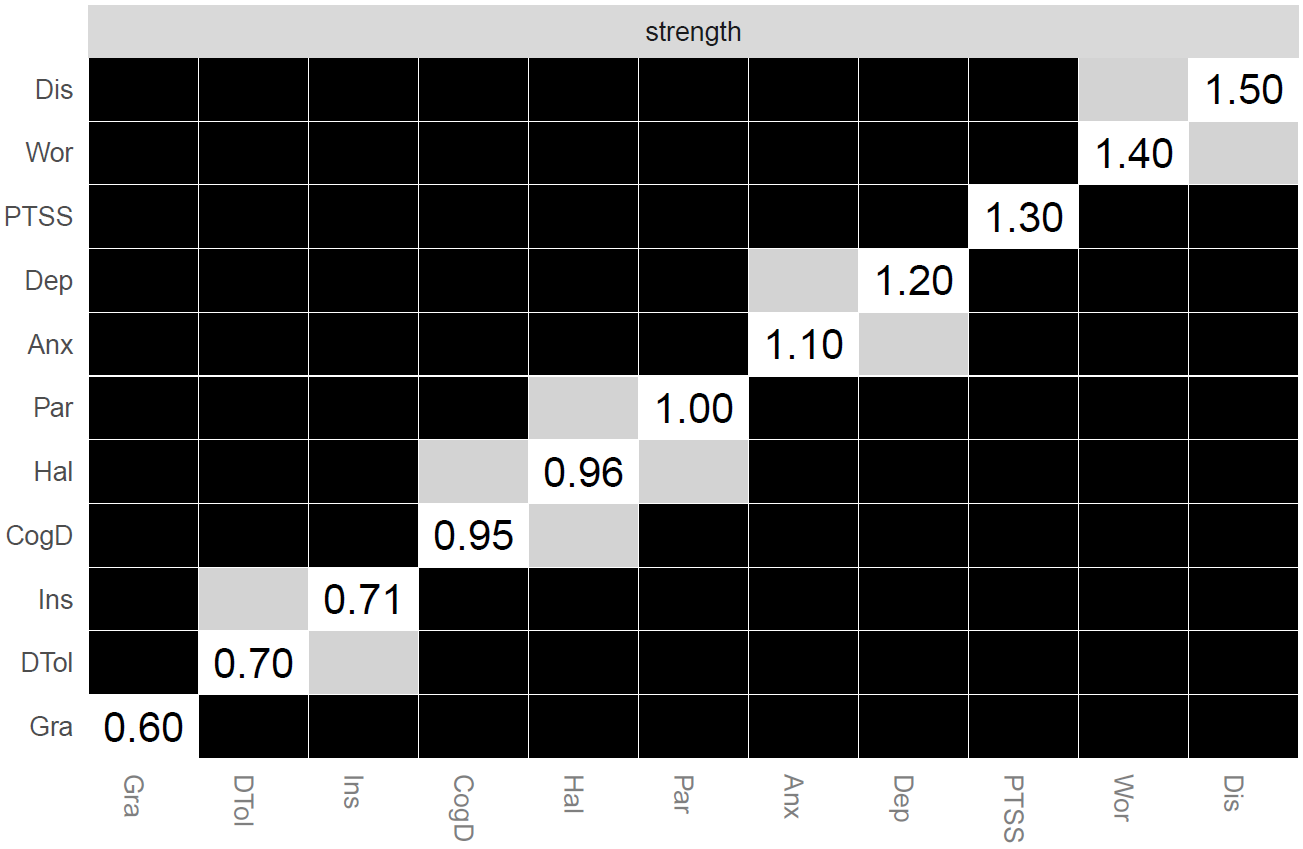


*Panel 4b: Closeness*


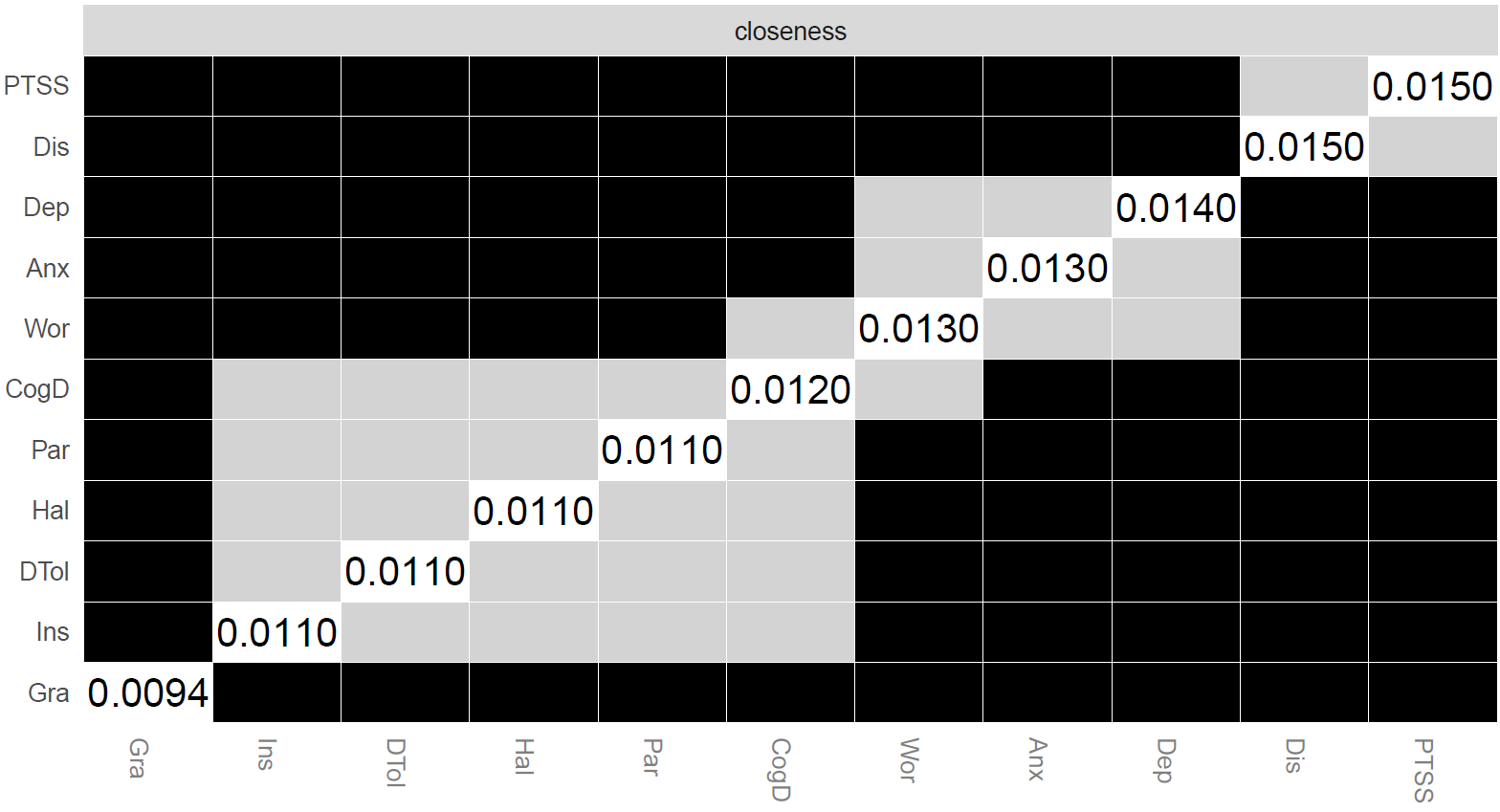


*Panel 4c: Betweenness*


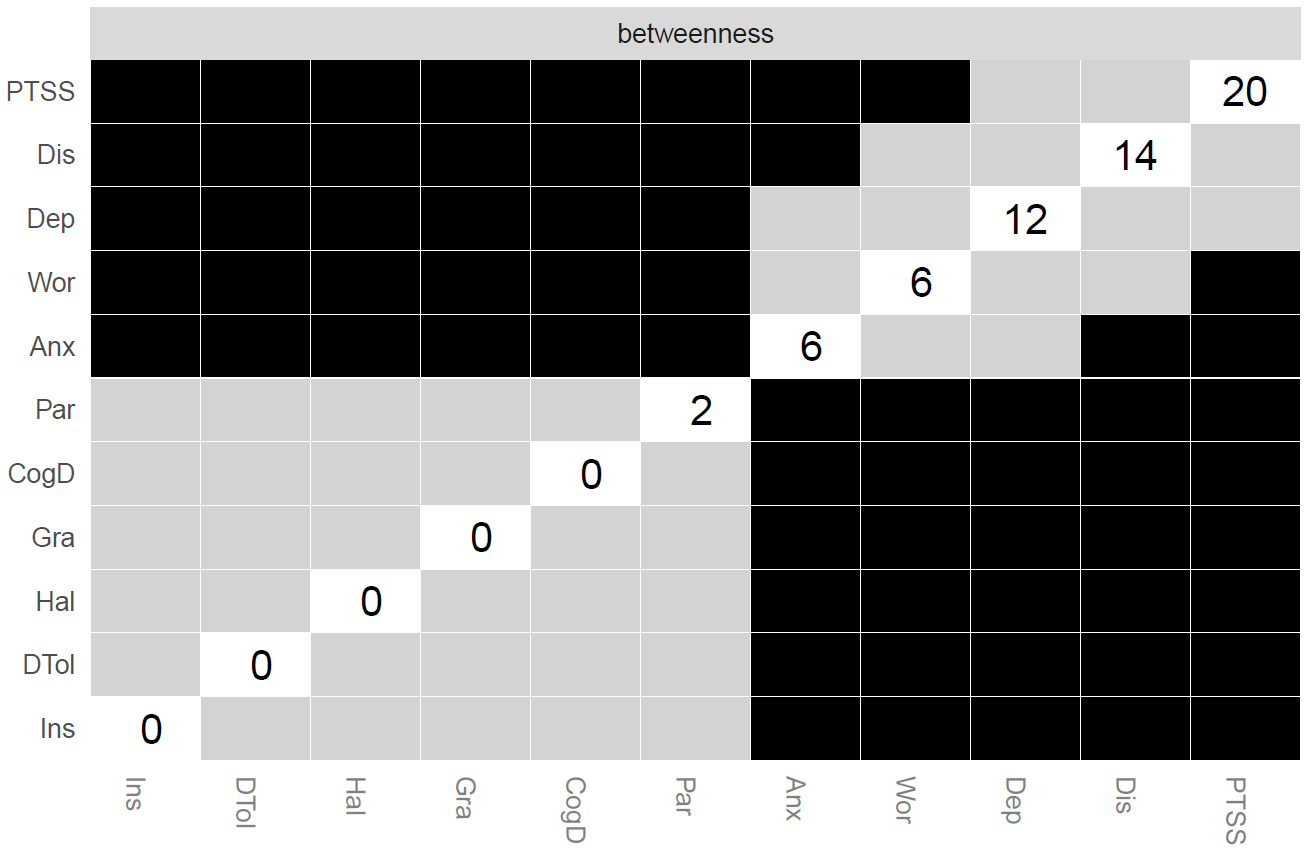


The stability of centrality estimates was calculated using case-dropping subset bootstrapping (Figure s5). This indicated that the stability for all three centrality measures was good. Correlation stability coefficients for all centrality estimates were 0.75, which meets the recommendation that this figure should be above 0.50.

***Figure s5.*** *Showing the results of the case-drop subset bootstrapping*


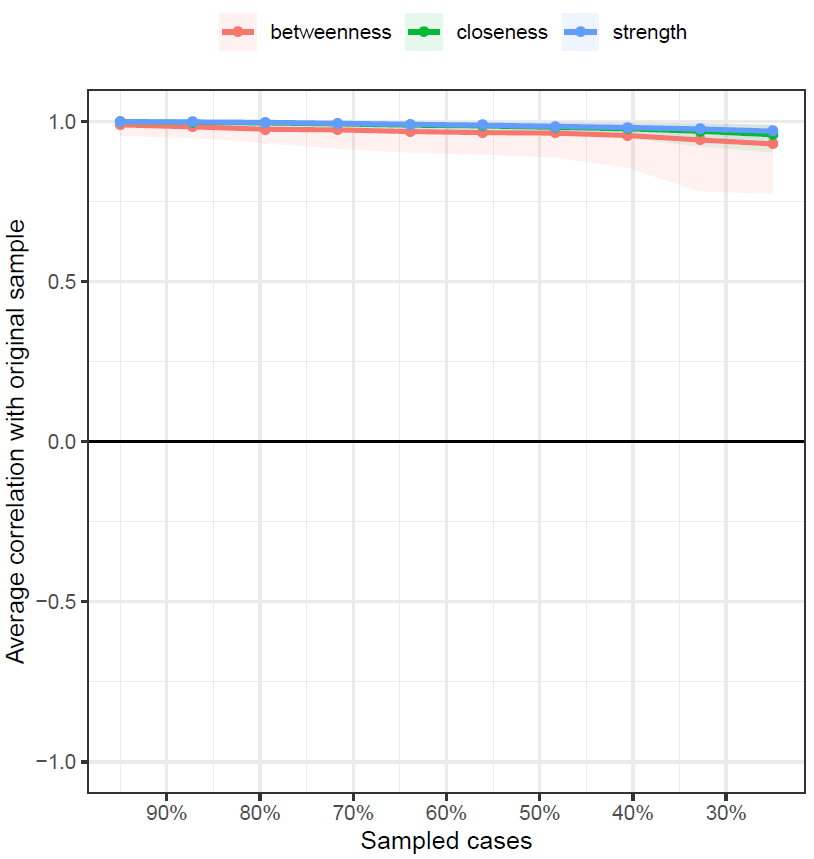


**SUPPLEMENTARY MATERIAL:
Correlation matrix and R-Code**

1. **Correlation matrix – Undirected network (Data fitted to Normal Distribution)**

|  | **Dis** | **Hal** | **PTSS** | **CogD** | **Dep** | **Par** | **Anx** | **Wor** | **Gra** | **Ins** | **DTol** |
| --- | --- | --- | --- | --- | --- | --- | --- | --- | --- | --- | --- |
| **Dis** | 1 | 0.6099 | 0.7064 | 0.6375 | 0.6126 | 0.6427 | 0.6249 | 0.4537 | 0.1677 | 0.4431 | -0.5032 |
| **Hal** | 0.6099 | 1 | 0.5149 | 0.4915 | 0.3958 | 0.5523 | 0.4433 | 0.3171 | 0.2068 | 0.3762 | -0.3603 |
| **PTSS** | 0.7064 | 0.5149 | 1 | 0.6059 | 0.6562 | 0.6393 | 0.6956 | 0.5410 | 0.0855 | 0.5740 | -0.5855 |
| **CogD** | 0.6375 | 0.4915 | 0.6059 | 1 | 0.5525 | 0.5781 | 0.5808 | 0.5644 | 0.0797 | 0.4274 | -0.5590 |
| **Dep** | 0.6126 | 0.3958 | 0.6562 | 0.5525 | 1 | 0.5039 | 0.6237 | 0.4679 | -0.0517 | 0.5312 | -0.4566 |
| **Par** | 0.6427 | 0.5523 | 0.6393 | 0.5781 | 0.5039 | 1 | 0.5987 | 0.5282 | 0.1894 | 0.4205 | -0.5079 |
| **Anx** | 0.6249 | 0.4433 | 0.6956 | 0.5808 | 0.6237 | 0.5987 | 1 | 0.7047 | 0.0443 | 0.4955 | -0.5506 |
| **Wor** | 0.4537 | 0.3171 | 0.5410 | 0.5644 | 0.4679 | 0.5282 | 0.7047 | 1 | -0.0153 | 0.4184 | -0.5742 |
| **Gra** | 0.1677 | 0.2068 | 0.0855 | 0.0797 | -0.0517 | 0.1894 | 0.0443 | -0.0153 | 1 | 0.0053 | -0.0479 |
| **Ins** | 0.4431 | 0.3762 | 0.5740 | 0.4274 | 0.5312 | 0.4205 | 0.4955 | 0.4184 | 0.0053 | 1 | -0.3683 |
| **DTol** | -0.5032 | -0.3603 | -0.5855 | -0.5590 | -0.4566 | -0.5079 | -0.5506 | -0.5742 | -0.0479 | -0.3683 | 1 |

1. **R-Code**

#### packages ####

library(tidyr)

library(dplyr)

library(qgraph)

library(bootnet)

library(DAGtools)

#### Undirected network ####

## Read & organise data

dat <- read.csv("Imputed 1.csv")

dat <- dat[ -c(1)] #remove ID

names(dat)[1] <- "Dis"

names(dat)[2] <- "Hal"

names(dat)[3] <- "PTSS"

names(dat)[4] <- "CogD"

names(dat)[5] <- "Dep"

names(dat)[6] <- "Par"

names(dat)[7] <- "Anx"

names(dat)[8] <- "Wor"

names(dat)[9] <- "Gra"

names(dat)[10] <- "Ins"

names(dat)[11] <- "DTol"

## fit data to Normal distribution

dat <- gaussianize(dat)

## Correlations

cor <- data.frame(cor(dat, use="pairwise.complete.obs"))

write.csv(cor, "Table-Correlation_matrix.csv", row.names = F)

## Estimate network

#create names

names <- c("Dissociation", "Hallucinations", "Post-Traumatic Stress Symptoms", "Cognitive Disorganisation", "Depression", "Paranoia", "Anxiety", "Worry", "Grandiosity", "Insomnia", "Distress Tolerance")

#run network analysis

undirectedNetwork <- estimateNetwork(dat, default = "ggmModSelect", principalDirection = TRUE)

#inspect undirected network as a graph

plot(undirectedNetwork, cut = 0.2, legend = T, nodeNames = names, legend.cex = 0.45)

##Assess accuracy and stability via bootstrapping

set.seed(123)

nBoots <- 5e3

nCores <- 8

Boot_cis <- bootnet(undirectedNetwork, nBoots = nBoots, nCores = nCores)

##EDGES & ACCURACY

# Figure of bootstrapped confidence intervals of the edge weights

pdf("Figure-Edge_weights_with_CIs.pdf", width = 7, height = 7)

plot(Boot_cis, order = "sample", plot = "interval", split0 = TRUE)

dev.off()

# Inspect edge weights with bootstrapped confidence intervals

ci <- as.data.frame(summary(Boot_cis))

Hal <- ci[ci$id=="Dis--Hal",]

PTSS <- ci[ci$id=="Dis--PTSS",]

CogD <- ci[ci$id=="Dis--CogD",]

Dep <- ci[ci$id=="Dis--Dep",]

Par <- ci[ci$id=="Dis--Par",]

Anx <- ci[ci$id=="Dis--Anx",]

Wor <- ci[ci$id=="Dis--Wor",]

Gra <- ci[ci$id=="Dis--Gra",]

Ins <- ci[ci$id=="Dis--Ins",]

DTol <- ci[ci$id=="Dis--DTol",]

edges <- c(Hal$sample, PTSS$sample, CogD$sample, Dep$sample, Par$sample, Anx$sample,Wor$sample,Gra$sample, Ins$sample, DTol$sample)

lowerCI <- c(Hal$CIlower, PTSS$CIlower, CogD$CIlower, Dep$CIlower, Par$CIlower, Anx$CIlower,Wor$CIlower,Gra$CIlower, Ins$CIlower, DTol$CIlower)

upperCI <- c(Hal$CIupper, PTSS$CIupper, CogD$CIupper, Dep$CIupper, Par$CIupper, Anx$CIupper,Wor$CIupper,Gra$CIupper, Ins$CIupper, DTol$CIupper)

table <- tibble(Variables = names[-1])

table2 <- table %>%

mutate(edges = edges, lowerCI = lowerCI, upperCI = upperCI)

write.csv(table2, "Table-Edge_weights_with_CIs.csv", row.names = F)

# Figure of edge weight differences

pdf("Figure-Edge_differences.pdf", width = 7, height = 7 * 2/3)

plot(Boot_cis, statistics = "edge", plot = "difference", onlyNonZero = T)

dev.off()

##CENTRALITY INDICES & STABILITY

cent <- centrality(undirectedNetwork)

round(cent$OutDegree,4)

round(cent$Closeness,5)

cent$Betweenness

# Figure showing centrality indices

pdf("Figure-Centrality.pdf", width = 7, height = 7 * 2/3)

centralityPlot(undirectedNetwork, include = c("Strength", "Closeness", "Betweenness"), labels = names)

dev.off()

# Bootstrap to test stability of centrality estimates

set.seed(123)

Boot_centrality <- bootnet(undirectedNetwork, nBoots = nBoots, default = "ggmModSelect", nCores = nCores, statistics=c("strength", "closeness", "betweenness"))

# Figures of centrality differences

pdf("Figure-Strength_centrality_difference.pdf", width = 5, height = 5 * 2/3)

plot(Boot_centrality, order = ‘sample’, statistics = c("strength"), plot = "difference", theme_bw=TRUE)

dev.off()

pdf("Figure-Closeness_centrality_difference.pdf", width = 6, height = 5 * 2/3)

plot(Boot_centrality, order = ‘sample’, statistics = c("closeness"), plot = "difference", theme_bw=TRUE)

dev.off()

pdf("Figure-Betweenness_centrality_difference.pdf", width = 5, height = 5 * 2/3)

plot(Boot_centrality, order = ‘sample’, statistics = c("betweenness"), plot = "difference", theme_bw=TRUE)

dev.off()

# Assess stability via case drop bootstrap

set.seed(123)

Boot_drop <- bootnet(undirectedNetwork, nBoots = nBoots, default = "ggmModSelect", nCores = nCores, type = "case", statistics = c("strength", "closeness", "betweenness"))

# coefficient of stability measures

cor_stab <- corStability(Boot_drop)

# Figure for the case drop results

pdf("Figure-Casedrop.pdf", width = 5, height = 6 * 2/3)

plot(Boot_drop, statistics = c("strength", "closeness", "betweenness"))

dev.off()

#### Directed network ####

## Read & organise data

dat1 <- read.csv("Imputed 1.csv")

dat2 <- read.csv("Imputed 2.csv")

dat3 <- read.csv("Imputed 3.csv")

dat4 <- read.csv("Imputed 4.csv")

dat5 <- read.csv("Imputed 5.csv")

dat1 <- dat1[-c(1)] #remove IDs

dat2 <- dat2[-c(1)]

dat3 <- dat3[-c(1)]

dat4 <- dat4[-c(1)]

dat5 <- dat5[-c(1)]

## fit data to Normal distribution

dag1 <- gaussianize(dat1)

dag2 <- gaussianize(dat2)

dag3 <- gaussianize(dat3)

dag4 <- gaussianize(dat4)

dag5 <- gaussianize(dat5)

mcmc_its <- 1e7

## estimate DAG - run MCMC

out <- DAGtools::fit_multiple(list(dag1, dag2, dag3, dag4, dag5), scoretype = "bge", iterations= mcmc_its)

##causal pathways for Table 2

sink(file = "causal_paths.out")

DAGtools::sum_causal_paths(out,1,2, digits = 4)

DAGtools::sum_causal_paths(out,1,3, digits = 4)

DAGtools::sum_causal_paths(out,1,4, digits = 4)

DAGtools::sum_causal_paths(out,1,5, digits = 4)

DAGtools::sum_causal_paths(out,1,6, digits = 4)

DAGtools::sum_causal_paths(out,1,7, digits = 4)

DAGtools::sum_causal_paths(out,1,8, digits = 4)

DAGtools::sum_causal_paths(out,1,9, digits = 4)

DAGtools::sum_causal_paths(out,1,10, digits = 4)

DAGtools::sum_causal_paths(out,1,11, digits = 4)

sink()

#### Plotting Graphs ####

## Figure 2 - DAGs with directed (>=90%) and undirected (>=50%) edges

A <- structure(c(0, 0.263936063936064, 0.0605394605394605, 0.248351648351648,

0.0047952047952048, 0, 0.151248751248751, 0.10969030969031, 0.000999000999000999,

0.156643356643357, 0.511488511488511,

0.736063936063936, 0, 0.0001998001998002,

0.142257742257742, 0.0915084915084915, 0.002997002997003, 0.0103896103896104,

0.166633366633367, 0, 0.234365634365634, 0.724875124875125, 0.939460539460539,

0.00699300699300699, 0, 0.973426573426573, 0.128871128871129,

0.58981018981019, 0.981818181818182, 0.00559440559440559, 0.0801198801198801,

0.982017982017982, 0.976623376623377, 0.554845154845155, 0.0255744255744256,

0.0265734265734266, 0, 0.11968031968032, 0.0025974025974026,

0.024975024975025, 0.169430569430569, 0.0025974025974026, 0.192007992007992,

0.702497502497503, 0.003996003996004, 0.908491508491509, 0.871128871128871,

0.88031968031968, 0, 0.002997002997003, 0.902297702297702, 0.022977022977023,

0.0021978021978022, 0.00999000999000999, 0.934865134865135, 0.00879120879120879,

0.997002997002997, 0.41018981018981, 0.997402597402597, 0.0037962037962038,

0, 0.998001998001998, 0.016983016983017, 0.0501498501498501,

0.964235764235764, 0.997402597402597, 0.848751248751249, 0.0371628371628372,

0.0181818181818182, 0.0271728271728272, 0.0531468531468531, 0.001998001998002,

0, 0.767232767232767, 0.0125874125874126, 0.179420579420579,

0.0385614385614386, 0.833366633366633, 0.833366633366633, 0.000599400599400599,

0.830569430569431, 0.000999000999000999, 0.0015984015984016,

0.232767232767233, 0, 0.0001998001998002, 0.211988011988012,

0.0387612387612388, 0.0163836163836164, 0.0041958041958042, 0.91988011988012,

0.00659340659340659, 0.0025974025974026, 0.94985014985015, 0.987412587412587,

0.0613386613386613, 0, 0.98981018981019, 0.191808191808192, 0.843356643356643,

0.765634365634366, 0.017982017982018, 0.806793206793207, 0.0193806193806194,

0.00819180819180819, 0.816583416583417, 0.788011988011988, 0.0101898101898102,

0, 0.861938061938062, 0.488511488511489, 0.275124875124875, 0.0233766233766234,

0.297502497502498, 0.0649350649350649, 0.0025974025974026, 0.0477522477522478,

0.0165834165834166, 0.0151848151848152, 0.138061938061938, 0), .Dim = c(11L,

11L),

.Dimnames = list(c("Anx", "Dep", "Par", "Hal", "Gra", "CogD",

"Wor", "Ins", "DTol", "PTSS", "Dis"),

c("Anx", "Dep", "Par", "Hal", "Gra", "CogD",

"Wor", "Ins", "DTol", "PTSS", "Dis")))

n_pos <- structure(c(-1.57819, 6.05767, -1.77872, 2.142, 6.38136, -0.7767,

-4.41439, 6.98681, -3.69857, 2.5585, 2.4256, 3.52285, 2.7593,

7.88781, 8.58053, 8.4895, -0.09931, 2.62501, -1.35754, -2.7,

-0.37396, 3.8), .Dim = c(11L, 2L),

.Dimnames = list(c("Anx", "Dep", "Par", "Hal", "Gra", "CogD", "Wor",

"Ins", "DTol", "PTSS", "Dis"), c("x", "y")))

TikzCode(A, file="plot_code_DAGs.tex", n_pos = n_pos, ud_thresh = 0.9, cutoff=0.5)

#This file (“plot_code_DAGs.tex”) then gets imported into a TeXworks script to build the final Figure

## Figure 1 – undirected network with the same layout

n_pos <- structure(c(2.4256, 2.142, 2.5585, -0.7767,

6.05767, -1.77872, -1.57819, -4.41439,

6.38136, 6.98681, -3.69857,

3.8,

8.58053, -0.37396, -0.09931, 2.7593, 7.88781,

3.52285, 2.62501, 8.4895, -1.35754,

-2.7), .Dim = c(11L, 2L),

.Dimnames = list(c("Dis", "Hal", "PTSS", "CogD", "Dep", "Par", "Anx", "Wor",

"Gra", "Ins", "DTol"), c("x","y")))

weight <- structure(c(0, 0.272500266665735, 0.249463157264878, 0.220270323226968,

0.191547866489037, 0.1579117887312, 0.138232421405264, -0.107489095063904,

0.103127607749572, -0.0574239106767876, 0, 0.272500266665735,

0, 0.0486911386626403, 0.115216180768117, -0.0467698748758263,

0.199335975989655, 0, -0.0728739490634346, 0.105429922512271,

0.101622694586213, 0, 0.249463157264878, 0.0486911386626403,

0, 0, 0.185502989271751, 0.148747562137055, 0.199199925128819,

0, 0, 0.230881030688042, 0.201457164103499, 0.220270323226968,

0.115216180768117, 0, 0, 0.13609208302436, 0.0957586069938397,

0, 0.197996149234388, 0, 0, 0.185106057117364, 0.191547866489037,

-0.0467698748758263, 0.185502989271751, 0.13609208302436, 0,

0, 0.192678110880923, -0.051387570480486, -0.174490573501693,

0.211425523976347, 0, 0.1579117887312, 0.199335975989655, 0.148747562137055,

0.0957586069938397, 0, 0, 0.0850703049907605, 0.131596750290918,

0.136374710878221, 0, 0.0702289225885052, 0.138232421405264,

0, 0.199199925128819, 0, 0.192678110880923, 0.0850703049907605,

0, 0.451623578169386, 0, 0.0432157341671571, 0, -0.107489095063904,

-0.0728739490634346, 0, 0.197996149234388, -0.051387570480486,

0.131596750290918, 0.451623578169386, 0, -0.0823962014896059,

0.0683974280691004, 0.240390275287333, 0.103127607749572, 0.105429922512271,

0, 0, -0.174490573501693, 0.136374710878221, 0, -0.0823962014896059,

0, 0, 0, -0.0574239106767876, 0.101622694586213, 0.230881030688042,

0, 0.211425523976347, 0, 0.0432157341671571, 0.0683974280691004,

0, 0, 0, 0, 0, 0.201457164103499, 0.185106057117364, 0, 0.0702289225885052,

0, 0.240390275287333, 0, 0, 0), .Dim = c(11L, 11L), .Dimnames = list(

c("Dis", "Hal", "PTSS", "CogD", "Dep", "Par", "Anx", "Wor",

"Gra", "Ins", "DTol"), c("Dis", "Hal", "PTSS", "CogD", "Dep",

"Par", "Anx", "Wor", "Gra", "Ins", "DTol")))

TikzCode(weight, z=weight,

file = "plot_code_ud.tex",

n_pos = n_pos,

col = c("blue", "red"),

nodesOnTop = TRUE, cutoff = 0, ud_thresh = 1, edgeScale = 2.5)

#This file (“plot_code_ud.tex”) then gets imported into a TeXworks script to build the final Figure
